# Formulation of the Alpha Sliding Innovation Filter: A Robust Linear Estimation Strategy

**DOI:** 10.3390/s22228927

**Published:** 2022-11-18

**Authors:** Mohammad AlShabi, Stephen Andrew Gadsden

**Affiliations:** 1 Department of Mechanical & Nuclear Engineering, University of Sharjah, Sharjah P.O. Box 27272, United Arab Emirates; 2 Department of Mechanical Engineering, McMaster University, Hamilton, ON L8S 4L8, Canada

**Keywords:** estimation theory, forgetting factor, Kalman filters, robustness, sliding innovation filter

## Abstract

In this paper, a new filter referred to as the alpha sliding innovation filter (ASIF) is presented. The sliding innovation filter (SIF) is a newly developed estimation strategy that uses innovation or measurement error as a switching hyperplane. It is a sub-optimal filter that provides a robust and stable estimate. In this paper, the SIF is reformulated by including a forgetting factor, which significantly improves estimation performance. The proposed ASIF is applied to several systems including a first-order thermometer, a second-order spring-mass-damper, and a third-order electrohydrostatic actuator (EHA) that was built for experimentation. The proposed ASIF provides an improvement in estimation accuracy while maintaining robustness to modeling uncertainties and disturbances.

## 1. Introduction

Estimation and filtering techniques are essential to successfully modeling and controlling engineering systems. By having a well-estimated state, the noise in the measured signal can be reduced and the quality of the controller can be improved [1]. Filters extract valuable information from noisy measurement signals and forward this information to a control system. Several works have studied filtering and estimation theory and can be summarized based on the principles that were used to develop the filter. Important features and methods that are considered for filters include optimality, robustness, and stability, combining techniques from the first two categories, and the utilization of artificial intelligence.

The most popular filter in the optimality category is the Kalman filter (KF), which was introduced by Rudolph Kalman in the 1960s [2]. This work was formulated to solve a linear stochastic system that involves Gaussian-white signals by reducing the state error covariance matrix. Later, a significantly large number of works were published that modified the KF in order to improve robustness and make it applicable to nonlinear systems and measurements. These works include the extended KF (EKF) [2,3], where a first-order Taylor series expansion is used to linearize the system and measurement matrices. Other works used the unscented transformation, such as the unscented KF (UKF) [3,4], and the Cubature rule, such as the cubature KF (CKF) [5], to approximate the nonlinear functions. These techniques used several points that are drawn from pre-defined density functions and propagated through the nonlinear functions. Next, the methods used statistical regression summation as a linearization technique.

Several works targeted the robustness of the KF by using simple algebraic techniques as in [6,7,8,9,10,11]. For example, some used QR decomposition and Cholesky factoring for the covariance matrices [10,11]. Other techniques used boundaries for the error to make sure it remained close to the state estimates [12]. For example, given the upper bounds on the level of modeling uncertainty, the KF gain may be bounded to help improve the estimation stability [12]. Other methodologies include the addition of fictitious system noise and adding a fading memory strategy [3,13]. Modifying the system noise matrix was performed when less confidence was placed in the system model used by the filter, or in other words when there was a great deal of system uncertainty [3,13,14,15]. Finally, the H∞ filter was introduced in [16,17]. This filter takes into consideration the worst-case uncertainties to derive the gain, which ensures that the estimates are bounded.

The second category targets the robustness and stability of the filter. The main filters in this area are sliding mode (SMO), variable structure (VSO) observers [13,18,19,20,21], and the strong tracking filter (STF) [22,23]. These observers define hyperplanes, usually functions of the innovation, and apply a discontinuous switching force to keep the estimate within a region of the true trajectory. For VSO, these hyperplanes divide the trajectory space into sub-regions in which the system dynamics are continuous. After crossing one of these planes, the observer changes its structure [20,24]. On the other hand, the hyperplanes of SMO are used to keep the estimates on them while sliding to towards zero [25,26]. A special form of the VSO and SMO was introduced in [1,27,28] in a predictor-corrector form, referred to as the smooth variable structure filter (SVSF). The SVSF gain is a function of the a priori and a posteriori innovation vectors and a memory function. Later, in 2020, a new filter referred to as the sliding innovation filter (SIF) joined the family [29]. SIF shared the same principles as the SVS but was simple in structure. SIF was used in target tracking [30,31], linear systems [32], unmanned aerial vehicles [33], and fault detection and diagnosis [34].

The third and fourth categories involve merging the first two categories to overcome limitations in each method, as per [35,36,37,38,39]. For example, the use of sigma points with the KF, such as central difference and unscented KF and the cubature KF, were combined with the SVSF in order to improve the accuracy of the SVSF while maintaining robustness. Other works combined the H∞ filter with the KF as in [40,41]. Some other works combined AI techniques such as in [42,43,44] where fuzzy logic was combined with the KF, and in [45,46,47] where AI was combined with the SMO and SVSF. The SIF was combined with multiple model-based filters in [48,49] and particle filters in [50].

In this paper, a novel algorithm referred to as the alpha sliding innovation filter (ASIF) is proposed. The proposed ASIF method gives an efficient yet simple mechanism that improves the performance of the standard SIF. By utilizing a forgetting factor coefficient, the level of measurement confidence can be optimized. Measurement noise affects the performance of the SIF; therefore, the SIF can be improved by minimizing the effects of noise using a forgetting factor. This paper is organized as follows. The SIF is introduced in Section 2, followed by the proposed ASIF algorithm in Section 3. The proof of stability for the proposed filter is derived in Section 4. The algorithm is tested and verified in Section 5 on three different systems. The paper is then concluded in Section 6.

## 2. The Sliding Innovation Filter (SIF)

The SIF is a new estimation technique that was proposed in [29] and later modified in [51,52]. The filter is a predictor-corrector filter that shares the same principles as the SVSF [35,53,54,55]. It utilizes the innovation as a switching hyperplane and forces the estimate to be within its neighborhood. The process starts by assuming that the system and measurements behave according to the following, respectively:

(1) $$x_{k+1}=Ax_{k}+Bu_{k}+w_{k}\;$$

(2) $$z_{k+1}=Cx_{k+1}+v_{k+1}\;$$

It then uses the knowledge of the system model to obtain a priori, predicted estimates and measurements as follows:

(3) $${\hat{x}}_{k+1|k}=A{\hat{x}}_{k|k}+Bu_{k}$$

(4) $${\tilde{z}}_{k+1|k}=z_{k+1}-C{\hat{x}}_{k+1|k}$$

The a priori estimate is refined in the corrector stage of the a posteriori estimate, as follows:

(5) $${\hat{x}}_{k+1|k+1}={\hat{x}}_{k+1|k}+K_{k+1}{\tilde{z}}_{k+1|k}$$

where $K_{k+1}$ is the correction gain of the SIF defined in Equation (6) and ${\tilde{z}}_{k+1|k}$ refers to the innovation or measurement error.

(6) $$K_{k+1}=C^{+}\bar{sat}\left(\left|{\tilde{z}}_{k+1|k}\right|/\delta \right)$$

where $\bar{sat}$ is the saturation function written as diagonal elements in a matrix and is defined as:

(7) $$\bar{sat}\left(\left|{\tilde{z}}_{k+1|k}\right|/\delta \right)=\left[\begin{array}{ccc} sat\left(\left|{\tilde{z}}_{1,k+1|k}\right|/\delta_{1}\right) & & \\ & \ddots & \\ & & sat\left(\left|{\tilde{z}}_{n,k+1|k}\right|/\delta_{n}\right) \end{array}\right]\;where\;sat\left(\left|A\right|/B\right)=\{\begin{array}{cc} 1 & \left|A\right|\geq B\\ \left|A\right|/B & \left|A\right|<B \end{array}$$

**Figure 1:**
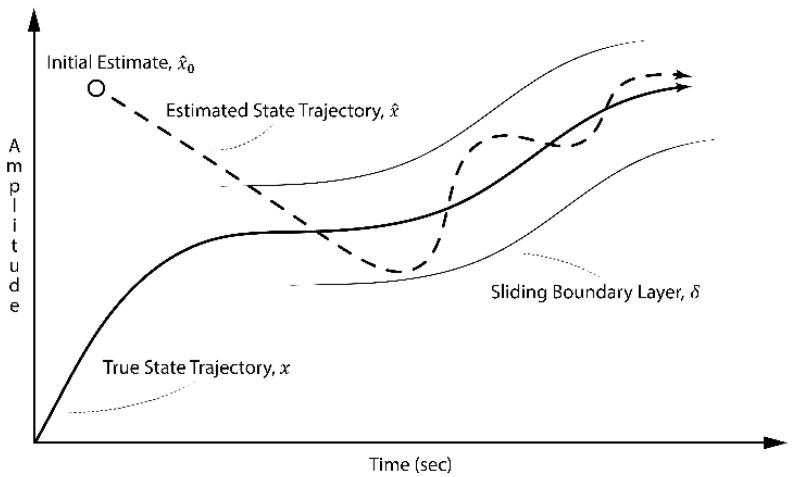
SIF estimation concept showing the sliding mode [29].

The elements have values between 0 and +1. The boundary layer δ is used to smooth the estimate and reduce the effect of the measurement noise [29]. However, if the innovation becomes larger than the boundary layer, then $\bar{sat}$ becomes the identity matrix. The SIF estimation process is illustrated in Figure 1.

## 3. The Proposed Alpha Sliding Innovation Filter (ASIF) Strategy

This section describes the proposed modification to the standard SIF. A forgetting factor coefficient (FF) or α is introduced to create the ASIF. The SIF using (2.6) is considered robust [29]. However, its performance in terms of optimality highly depends on the selection of the boundary layer. The selection mechanism proposed in [29] to obtain the widths of these layers was based on trial and error. However, this is not an easy task for several reasons, summarized as follows:

1. The range of acceptable δ is infinitely wide, as it can be any positive value (0,∞).
2. The number of boundary layers required is equal to the number of measurements, which means exhaustive trials for high-dimensional systems (e.g., AI output layers).
3. In most cases, δ is selected to be the maximum allowable error in the system. For small innovation amplitudes, the filter will have a very small convergence rate, and for large amplitudes, the estimates may chatter.

These limitations inspired the authors to develop a modified version of the SIF that improves the performance while overcoming these limitations. A fading memory is added to the SIF’s gain. By adding the coefficient α, the $\bar{sat}$ term can be ignored and replaced with an identity matrix. In our case, α is a measure of measurement confidence, as per the following:

1. If α=1, ASIF collapses to the SIF with a very small boundary layer.
2. If α→0, the filter depends more on the system and less on the measurement. This can be used to reduce the effect of the measurement noise. This helps significantly when R is larger than Q.
3. If α is large, the filter depends more on the measurement, which makes the ASIF sensitive to the measurement noise but less sensitive to modeling uncertainties. This helps when Q is larger than R.

For the ASIF estimation strategy, the proposed gain has the following simplified structure:

(8) $$K_{k+1}=\alpha C^{+}$$

Note that the ASIF estimation process is similar to the SIF, except that Equation (8) is used instead of Equation (6).

## 4. Proof of Stability

The SIF gain was developed to guarantee the stability and robustness of the filter. Adding the forgetting factor changes the filter structure. In this section, the stability of the new ASIF is examined. We consider the discrete Lyapunov function $M_{k+1}$ defined as follows:

(9) $$M_{k+1}=\left|{\tilde{z}}_{k+1|k+1}\right|$$

Then, according to Equation (9), the proposed filter is stable as long as the innovation is decreasing with time, as follows:

(10) $$\left|{\tilde{z}}_{k+1|k+1}\right|<\left|{\tilde{z}}_{k|k}\right|$$

Referring to the equations of Section 2 and Section 3, taking the difference between Equations (1) and (5) yields:

(11) $${\tilde{x}}_{k+1|k+1}={\tilde{x}}_{k+1|k}-\alpha C^{+}{\tilde{z}}_{k+1|k}$$

Substituting Equation (2) in (4) results in:

(12) $${\tilde{z}}_{k+1|k}=C{\tilde{x}}_{k+1|k}+v_{k+1}\;or\;C^{+}{\tilde{z}}_{k+1|k}={\tilde{x}}_{k+1|k}+C^{+}v_{k+1}$$

Substituting Equation (12) in (11) and multiplying by C gives:

(13) $${\tilde{z}}_{k+1|k+1}={\tilde{z}}_{k+1|k}-\alpha {\tilde{z}}_{k+1|k}\;or\;{\tilde{z}}_{k+1|k+1}=\left(1-\alpha \right){\tilde{z}}_{k+1|k}$$

Subtracting Equation (3) from Equation (1) and using Equation (12) yields the following:

(14) $${\tilde{x}}_{k+1|k}=A{\tilde{x}}_{k|k}+w_{k}$$

(15) $${\tilde{z}}_{k+1|k}=C\left(A{\tilde{x}}_{k|k}+w_{k}\right)+v_{k+1}$$

Substituting Equation (15) into Equation (13) yields:

(16) $${\tilde{z}}_{k+1|k+1}=\left(1-\alpha \right)\left(C\left(A{\tilde{x}}_{k|k}+w_{k}\right)+v_{k+1}\right)$$

Rewriting Equation (16) and simplifying yields:

(17) $${\tilde{z}}_{k+1|k+1}=\left(1-\alpha \right)CAC^{+}{\tilde{z}}_{k|k}+\eta$$

where η is defined as an uncertainty vector as follows:

(18) $$\eta =-\left(1-\alpha \right)C\left(AC^{+}v_{k}-w_{k}\right)+\left(1-\alpha \right)v_{k+1}$$

If the measurement and system noise vectors are white, then $E\left[w_{k}\right]=E\left[v_{k}\right]=E\left[\eta \right]=0$. Therefore, taking the expectation of Equation (17) yields:

(19) $$E\left[{\tilde{z}}_{k+1|k+1}\right]=\left(1-\alpha \right)CAC^{+}E\left[{\tilde{z}}_{k|k}\right]$$

From Equation (19) and Equation (20), the filter is considered stable if

(20) $$\left|\left(1-\alpha \right)CAC^{+}\right|<1$$

where E refers to the expectation or expected value. The system under study is stable if the eigenvalues of A are less than unity. Therefore, without losing generality, $CAC^{+}$ is less than unity, which yields the following condition:

(21) −1<(1−α)<1

Solving Equation (21) further gives:

(22) 0≤α≤2

Therefore, for the ASIF estimation process to be stable, α must be defined as per Equation (22).

## 5. Computer Experiments and Results

The proposed ASIF estimation strategy will be tested on first-, second-, and third-order systems. The ASIF is compared with the original SIF and the well-known KF. The study was conducted on MATLAB and was repeated 1000 times. The repetitions were conducted to obtain the Monte Carlo Simulation (MCS) in order to provide better insight into the different estimation methods. The parameters, Q, R, δ, and α are estimated for no modeling uncertainties case. Their values are obtained by trial and error. They are assumed to be valid for the molding uncertainties case.

The results are compared in terms of root mean squared error (*RMSE*) and maximum absolute error (*MAE*) under normal operating conditions, and modeling uncertainty or faulty conditions. *RMSE* and *MAE* are calculated using Equations (23) and (24), respectively, assuming n is the number of sampled data.

(23) $$RMSE=\sqrt{\frac{\sum_{i=1}^{n}{\left(x_{i}-{\hat{x}}_{i}\right)}^{2}}{n}}$$

(24) $$MAE=\max\left({\left|x_{i}-{\hat{x}}_{i}\right|}_{i=1\ldots n}\right)$$

### 5.1. Mercury Thermometer

A thermometer has a simple first-order differential equation, which is derived from the conservation of energy. The fluid transfers the heat by convection and stores it in the fluid using:

(25) $$-hA\Delta T=mC\frac{dT}{dt}$$

The above relation can be simplified to:

(26) $$\frac{dT}{dt}+\frac{hA_{p}}{mC_{p}}\Delta T=\frac{dT}{dt}+\frac{1}{\tau }\Delta T=0$$

where τ is the time constant with a value of 1.7 sec [56]. Rearranging Equation (26) yields:

(27) $$\frac{dT}{dt}+\frac{1}{\tau }T=\frac{1}{\tau }T_{i}$$

Equation (25) can be discretized into the following:

(28) $$T_{k+1}=\left(1-\frac{T_{s}}{\tau }\right)T_{k}+\left\{\frac{T_{s}}{\tau }T_{i,k}=u_{k}\right\}+w_{k}$$

or using the following state space form:

(29) $$x_{k+1}=\left[1-\frac{T_{s}}{\tau }\right]x_{k}+\left[\frac{T_{s}}{\tau }\right]u_{k}+w_{k}=Ax_{k}+Bu_{k}+w_{k}$$

(30) $$z_{k+1}=Cx_{k+1}+v_{k+1}$$

where u is the temperature of the surroundings and is treated as the system input. It is selected to change its value between 25 °C and 50 °C every second. The system and measurement noises (w and v) are normally distributed with zero mean and covariance’s Q and R defined by Equations (31) and (32), respectively. C is equal to unity and α is selected as shown in Equation (33).

(31) Q=0.5775

(32) R=0.5813

(33) α=0.6182

**Figure 2:**
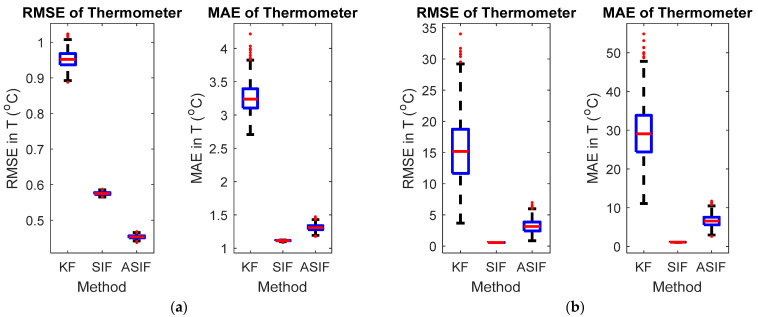
RMSE and MAE for thermometer with (**a**) no modeling and (**b**) modeling uncertainties.

**Figure 3:**
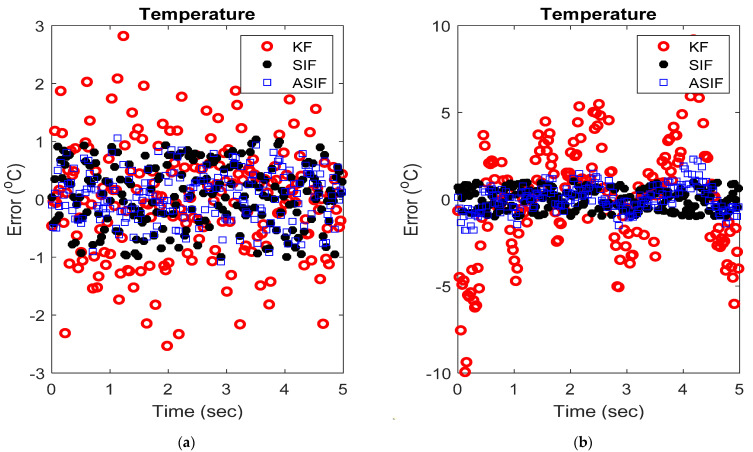
An example of the outputs from the different filters applied to the thermometer system with (**a**) no modeling and (**b**) modeling uncertainties.

The initial values for the filters and their corresponding initial covariance values were set to zero and one, respectively. The boundary layer was set to 0.5 based on trial and error in order to minimize the estimation error. The simulations were repeated after injecting the system with an uncertainty of 20%, i.e., A becomes 0.7995. Figure 2 shows the averaged RMSE and MAE for the system with and without modeling uncertainties, respectively. Figure 3 shows the actual estimates for the two respective cases. Table 1 and Table 2 include the calculated RMSE and MAE of the MCS without and with modeling uncertainties, respectively.

When no modeling uncertainties were present, the RMSE and MAE results for the ASIF illustrate a better performance compared with the KF and SIF strategies. The performance of the ASIF remains better than the KF when uncertainties were present. Compared to the standard SIF, the ASIF yielded better estimates. However, when uncertainties were present, both the SIF and ASIF estimation strategies performed similarly, with slight superiority to SIF.

### 5.2. Spring-Mass-Damper

The well-known spring-mass-damper system (S-M-D) is mathematically described using a second-order differential equation, according to the following formula:

(34) $$M\frac{d^{2}x}{dt^{2}}+b\frac{dx}{dt}+kx=u$$

Equation (34) can be represented as discretized state space equations (system and measurement) as follows:

(35) $$x_{k+1}=\left[\begin{array}{cc} 1 & T_{s}\\ -kT_{s}/M & \left(1-bT_{s}/M\right) \end{array}\right]x_{k}+\left[\begin{array}{c} 0\\ T_{s}/M \end{array}\right]u$$

(36) $$z_{k+1}=Cx_{k+1}+v_{k+1}$$

where u is the force applied to the system, which is selected to change its value between 50 and 500 Newton every second. The system and measurement noises (w and v) are normally distributed with zero mean and covariance’s Q and R defined by Equations (37) and (38), respectively. The boundary layer and α are chosen as shown in Equations (39) and (40), respectively. C is the identity matrix.

(37) $$Q=diag\left(\left[\begin{array}{cc} 2.3 & 2.3 \end{array}\right]\right)\times 10^{-14}$$

(38) $$R=diag\left(\left[\begin{array}{cc} 5.8 & 5.8 \end{array}\right]\right)\times 10^{-3}$$

(39) $$\psi ={\left[0.1\;0.01\right]}^{T}$$

(40) α=0.05

**Figure 4:**
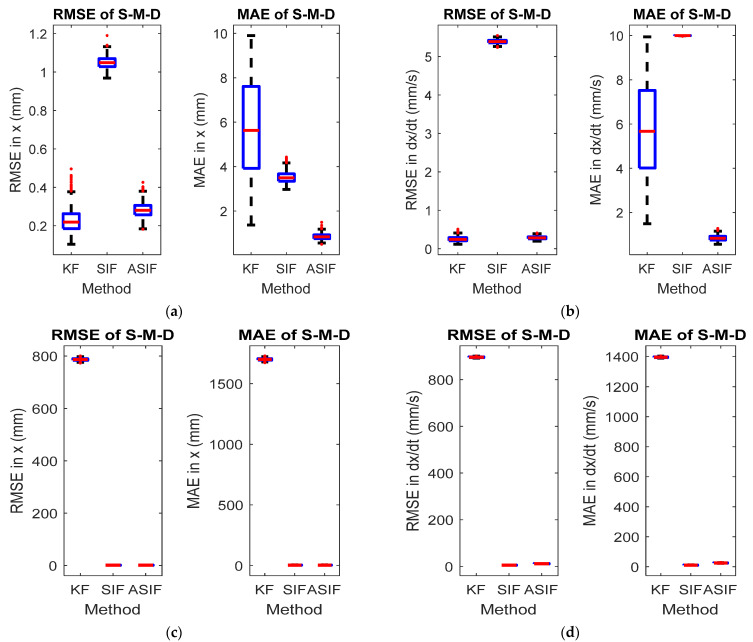
RMSE and MAE for the S-M-D system’s (**a**) first and (**b**) second states with no modeling uncertainties, and (**c**) first and (**d**) second states with modeling uncertainties.

**Figure 5:**
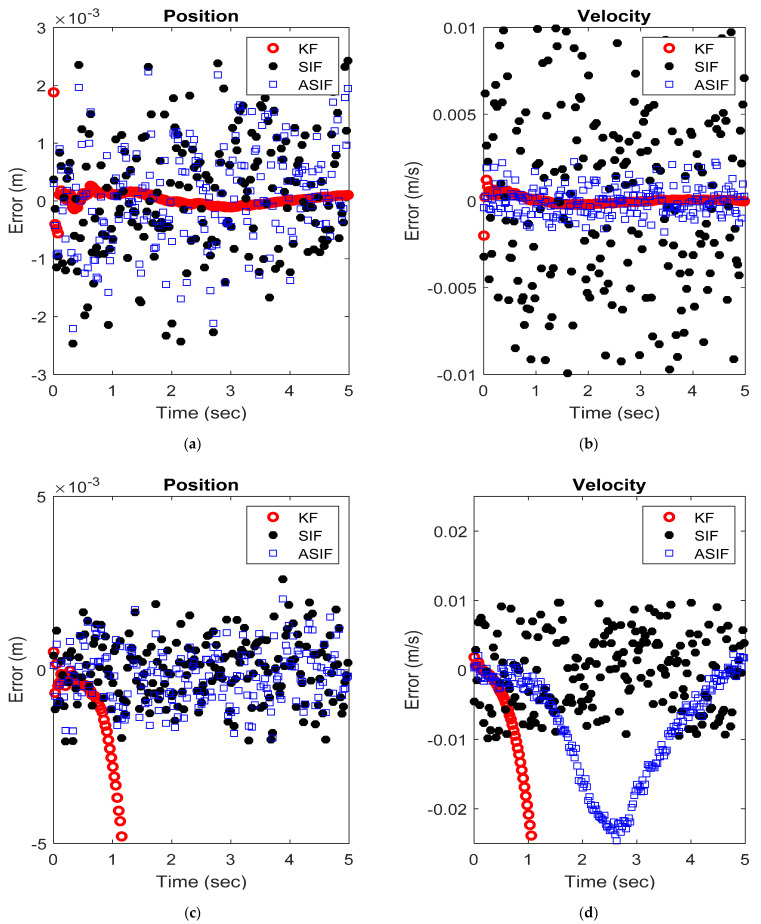
An example of the (**a**) position and (**b**) velocity estimates from the different filters applied to the S-M-D system with no modeling uncertainties, and (**c**) position and (**d**) velocity estimates from the different filters applied to the S-M-D system with modeling uncertainties.

The initial values for the filters and their corresponding initial covariance values were set to zero vectors and identity matrices, respectively. The simulations were repeated after injecting the system with uncertainties as mentioned in Table 3. The system parameters are summarized in Table 3. Figure 4 shows the RMSE and MAE for the first and second states for the two case scenarios: No modeling and modeling uncertainties present. The true and estimated state values for the first and second states with no modeling and with modeling uncertainties present are shown in Figure 5. Table 4 and Table 5 summarize the averaged RMSE and MAE for the cases without and with modeling uncertainties. ASIF shows a similar performance to KF when no modeling uncertainties present, which is better than those obtained by SIF. Once modeling uncertainties were injected, ASIF has a similar performance to SIF, which is better than KF.

### 5.3. Electrohydrostatic Actuator

The third and final example in this study includes an aerospace flight surface actuator proposed and studied in [6,27,29]. The linear system and measurements are formulated as:

(41) $$x_{k+1}=\left[\begin{array}{ccc} 1 & T_{s} & 0\\ 0 & 1 & T_{s}\\ -557 & -28.6 & 0.94 \end{array}\right]x_{k}+\left[\begin{array}{c} 0\\ 0\\ 557 \end{array}\right]u_{k}+w_{k}$$

(42) $$z_{k+1}=Cx_{k+1}+v_{k+1}$$

where u is the controller input. In this case, it is defined as a multistep input that changes its value between −0.5 and 0.5 rad/s every second. The system and measurement noise covariance’s are Q and R defined by Equations (43) and (44), respectively. As per the previous examples, modeling uncertainties are injected using Equation (45). The boundary layer and α are chosen as shown in Equations (46) and (47), respectively. C is the identity matrix.

(43) $$Q=diag\left(\left[\begin{array}{ccc} 1.8\times 10^{-2} & 1.8\times 10^{-2} & 1.8\times 10^{-2} \end{array}\right]\right)$$

(44) $$R=diag\left(\left[\begin{array}{ccc} 5.8 & 5.8 & 5.8 \end{array}\right]\right)\times 10^{-2}$$

(45) $$x_{k+1}=\left[\begin{array}{ccc} 1 & T & 0\\ 0 & 1 & T\\ -55.7 & -2.86 & 0.97 \end{array}\right]x_{k}+\left[\begin{array}{c} 0\\ 0\\ 55.7 \end{array}\right]u_{k}+w_{k}$$

(46) $$\psi ={\left[0.1\;0.5\;0.5\right]}^{T}$$

(47) α=0.9995

**Figure 6:**
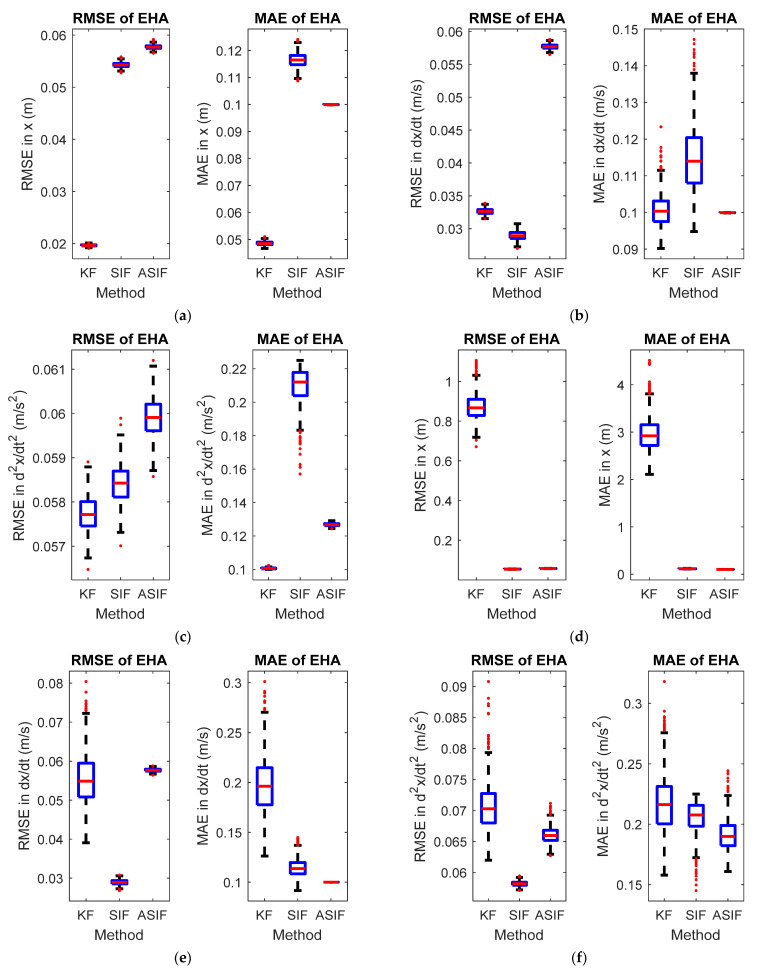
RMSE and MAE for the EHA system’s (**a**) first, (**b**) second, and (**c**) third states with no modeling uncertainties, and the system’s (**d**) first, (**e**) second, and (**f**) third states with modeling uncertainties.

**Figure 7:**
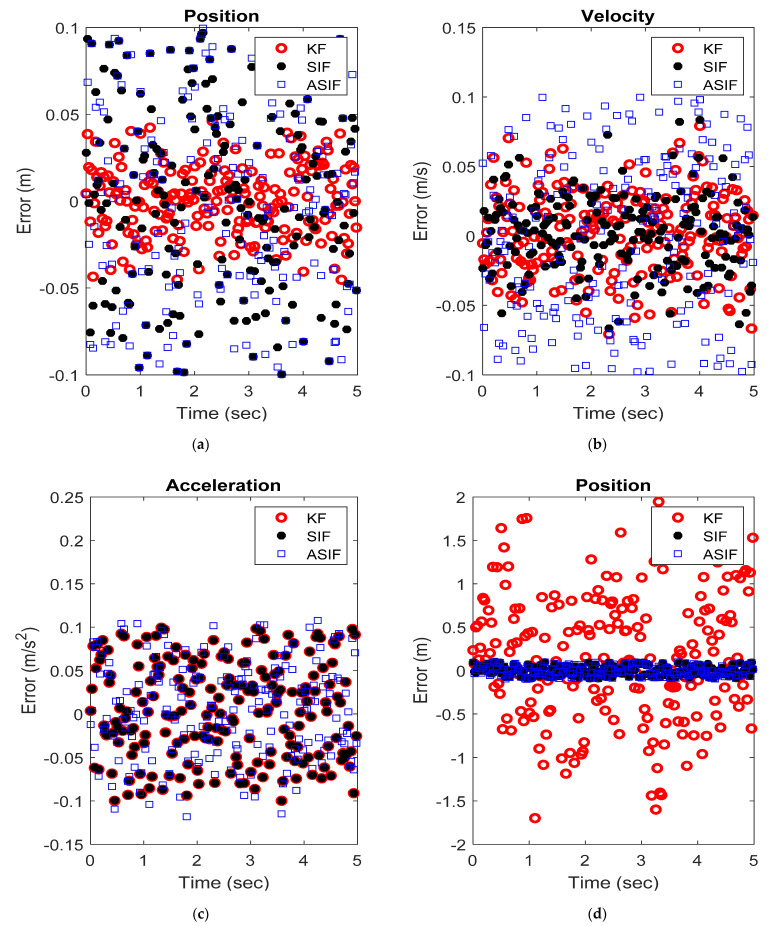

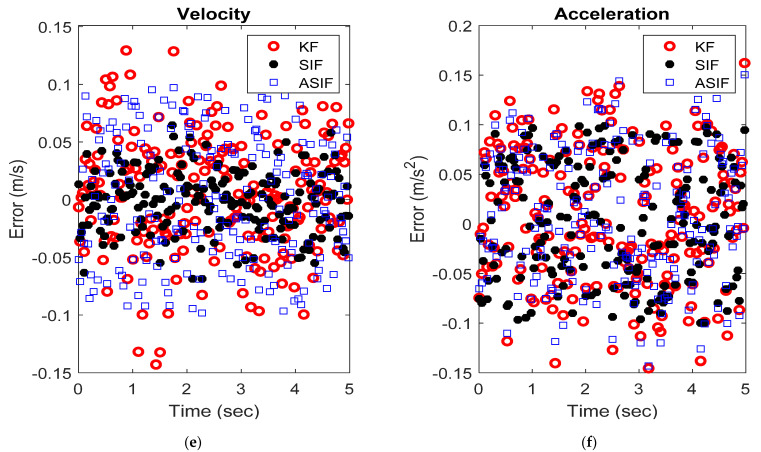
An example of the (**a**) position, (**b**) velocity, and (**c**) acceleration estimates from the different filters applied to the aerospace actuator system with no modeling uncertainties, and the (**d**) position, (**e**) velocity, and (**f**) acceleration estimates from the different filters applied to the aerospace actuator system with modeling uncertainties.

The initial values for the filters, their corresponding initial covariance values, and the boundary layer’s width were set to be similar to the S-M-D example. Figure 6 shows the RMSE and MAE for the three states without and with modeling uncertainties present. Figure 7 shows the actual and estimated state values for the states in both scenarios. Table 6 and Table 7 summarize the RMSE and MAE for the cases without and with modeling uncertainties.

Under normal operating conditions, the KF provided the best results (the RMSE and MAE were the smallest). In the presence of uncertainties (e.g., a fault) and after tuning the KF and ASIF, they performed similarly. Note that the SIF and ASIF were not as sensitive to uncertainties compared with the KF.

**Figure 8:**
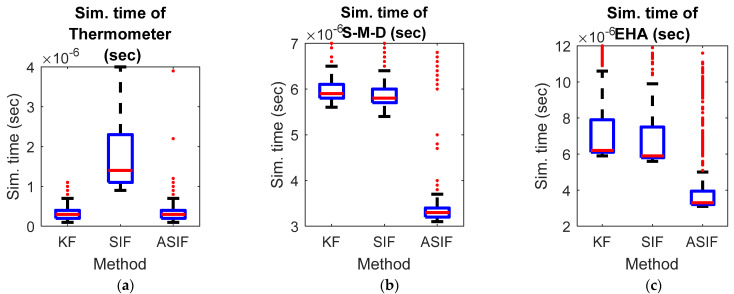
The simulation time for (**a**) thermometer, (**b**) spring-mass-damper, and (**c**) EHA examples for the different filters.

To complete the comparison, the simulation time for the three previous examples was measured for the three filters, namely KF, SIF, and ASIF. Figure 8 shows that the ASIF has the shortest time among the other filters. It requires less than half the time that is required for the other filters in the second and third examples. Adding this to the results obtained from RMSE and MAE tables, ASIF can be considered to have an overall superior performance.

## 6. Conclusions

In this paper, a new linear filter referred to as the ASIF is presented. The ASIF takes advantage of the robustness and stability of the sliding innovation filter (SIF) while allowing for a design parameter, which is a measure of our confidence in the measurement. The addition of a so-called forgetting factor improves the performance of the standard SIF while maintaining robustness to modeling uncertainties. Furthermore, the tuning process for the design parameter is significantly easier than defining the sliding boundary layer widths used by the standard SIF. ASIF needs less computational time compared to the other filter, which makes it the best candidate for fast online systems. Future work will include the development of a time-varying forgetting factor to create an adaptive ASIF and an optimal ASIF.

## Figures and Tables

**Table 1:** RMSE For MCS of The Thermometer Example.

Case	State	KF		SIF		ASIF	
		Mean	σ	Mean	σ	Mean	σ
1	$x_{1}$	$9.5\times 10^{-1}$	$2.2\times 10^{-2}$	$5.8\times 10^{-1}$	$3.7\times 10^{-3}$	$4.5\times 10^{-1}$	$4.6\times 10^{-3}$
2	$x_{1}$	$2.9\times 10^{1}$	$7.1\times 10^{0}$	$1.1\times 10^{0}$	$2.5\times 10^{-2}$	$6.6\times 10^{0}$	$1.5\times 10^{0}$

**Table 2:** MAE for MCS of the thermometer example.

Case	State	KF		SIF		ASIF	
		Mean	σ	Mean	σ	Mean	σ
1	$x_{1}$	$3.3\times 10^{0}$	$2.2\times 10^{-1}$	$1.1\times 10^{0}$	$6.8\times 10^{-3}$	$1.3\times 10^{0}$	$4.4\times 10^{-2}$
2	$x_{1}$	$9.998\times 10^{-1}$	$4.99\times 10^{1}$	$1.4\times 10^{0}$	$3.6\times 10^{-3}$	$1.0\times 10^{0}$	$1.0\times 10^{0}$

**Table 3:** Parameters Used In the S-M-D Example.

Parameter	Case 1	Case 2
M (kg)	500	500
k (kN/m)	1	0.5
b (Ns/m)	5	0

**Table 4:** RMSE For MCS Of The S-M-D Example.

Case	State	KF		SIF		ASIF	
		Mean	σ	Mean	σ	Mean	σ
1	$x_{1}$	$2.0\times 10^{-4}$	$6.1\times 10^{-5}$	$1.0\times 10^{-3}$	$3.2\times 10^{-5}$	$3.0\times 10^{-4}$	$3.8\times 10^{-5}$
	$x_{2}$	$3.0\times 10^{-4}$	$6.4\times 10^{-5}$	$5.4\times 10^{-3}$	$4.5\times 10^{-5}$	$3.0\times 10^{-4}$	$4.0\times 10^{-5}$
2	$x_{1}$	$7.9\times 10^{-1}$	$4.0\times 10^{-3}$	$1.0\times 10^{-3}$	$0.0\times 10^{0}$	$9.0\times 10^{-4}$	$0.0\times 10^{0}$
	$x_{2}$	$8.9\times 10^{-1}$	$1.3\times 10^{-3}$	$5.4\times 10^{-3}$	$0.0\times 10^{0}$	$1.2\times 10^{-2}$	$1.0\times 10^{-4}$

**Table 5:** MAE for MCS of the S-M-D example.

Case	State	KF		SIF		ASIF	
		Mean	σ	Mean	σ	Mean	σ
1	$x_{1}$	$5.8\times 10^{-3}$	$2.3\times 10^{-3}$	$3.5\times 10^{-3}$	$3.0\times 10^{-4}$	$8.0\times 10^{-4}$	$1.0\times 10^{-4}$
	$x_{2}$	$5.8\times 10^{-3}$	$2.2\times 10^{-3}$	$1.0\times 10^{-2}$	$0.0\times 10^{0}$	$9.0\times 10^{-4}$	$1.0\times 10^{-4}$
2	$x_{1}$	$1.7\times 10^{0}$	$8.1\times 10^{-3}$	$3.5\times 10^{-3}$	$3.0\times 10^{-4}$	$3.3\times 10^{-3}$	$3.0\times 10^{-4}$
	$x_{2}$	$1.4\times 10^{0}$	$2.0\times 10^{-3}$	$1.1\times 10^{-2}$	$3.0\times 10^{-4}$	$2.5\times 10^{-2}$	$5.0\times 10^{-4}$

**Table 6:** RMSE for MCS of the Aerospace Actuator Example.

Case	State	KF		SIF		ASIF	
		Mean	σ	Mean	σ	Mean	σ
1	$x_{1}$	$2.0\times 10^{-2}$	$1.7\times 10^{-4}$	$5.4\times 10^{-2}$	$4.4\times 10^{-4}$	$5.8\times 10^{-2}$	$3.6\times 10^{-4}$
	$x_{2}$	$3.3\times 10^{-2}$	$4.2\times 10^{-4}$	$2.9\times 10^{-2}$	$6.3\times 10^{-4}$	$5.8\times 10^{-2}$	$3.6\times 10^{-4}$
	$x_{3}$	$5.8\times 10^{-2}$	$3.9\times 10^{-4}$	$5.8\times 10^{-2}$	$4.3\times 10^{-4}$	$5.99\times 10^{-2}$	$4.3\times 10^{-4}$
2	$x_{1}$	$8.7\times 10^{-1}$	$6.7\times 10^{-2}$	$5.4\times 10^{-2}$	$5.0\times 10^{-4}$	$5.8\times 10^{-2}$	$4.0\times 10^{-4}$
	$x_{2}$	$5.5\times 10^{-2}$	$6.5\times 10^{-3}$	$2.9\times 10^{-2}$	$6.0\times 10^{-4}$	$5.8\times 10^{-2}$	$4.0\times 10^{-4}$
	$x_{3}$	$7.1\times 10^{-2}$	$3.7\times 10^{-3}$	$5.8\times 10^{-2}$	$4.0\times 10^{-4}$	$6.6\times 10^{-2}$	$1.3\times 10^{-3}$

**Table 7:** MAE for MCS of the Aerospace Actuator Example.

Case	State	KF		SIF		ASIF	
		Mean	σ	Mean	σ	Mean	σ
1	$x_{1}$	$4.9\times 10^{-2}$	$7.0\times 10^{-4}$	$1.2\times 10^{-1}$	$2.5\times 10^{-3}$	$9.99\times 10^{-2}$	$0.0\times 10^{0}$
	$x_{2}$	$1.0\times 10^{-1}$	$4.5\times 10^{-3}$	$1.1\times 10^{-1}$	$9.2\times 10^{-3}$	$9.99\times 10^{-2}$	$0.0\times 10^{0}$
	$x_{3}$	$1.0\times 10^{-1}$	$4.0\times 10^{-4}$	$2.1\times 10^{0}$	$1.1\times 10^{-2}$	$1.3\times 10^{-1}$	$0.0\times 10^{0}$
2	$x_{1}$	$2.95\times 10^{0}$	$3.4\times 10^{-1}$	$1.1\times 10^{-1}$	$2.7\times 10^{-3}$	$9.99\times 10^{-2}$	$0.0\times 10^{0}$
	$x_{2}$	$1.98\times 10^{-1}$	$2.8\times 10^{-2}$	$1.1\times 10^{-1}$	$9.1\times 10^{-3}$	$9.99\times 10^{-2}$	$0.0\times 10^{0}$
	$x_{3}$	$2.2\times 10^{-1}$	$2.4\times 10^{-2}$	$2.1\times 10^{-1}$	$1.3\times 10^{-2}$	$1.9\times 10^{-1}$	$1.3\times 10^{-2}$

## Data Availability

No new data were created or analyzed in this study. Data sharing is not applicable to this article.

## Nomenclature

**Symbol**	**Comments**
${}^{-1}$ $,\;{}^{+}$ $,\;{}^{T}$	Notation denoting an inverse, a pseudo inverse and a transpose, respectively.
|A|	Absolute value of A.
A, B	The system, and input matrices, respectively.
C	The measurement matrix, which is the identity matrix in all examples.
$w_{k},\;v_{k}$	The system and measurement noise vectors at time k, respectively.
$x_{k},\;z_{k},\;u_{k}$	The states, measurements and imput vectores, respectively.
$\hat{A}$	The estimate value of A.
$K_{k}$	The correction matrix at time *k*.
δ	The Boundary Layer
$A_{k|k-1},\;A_{k|k}$	The a priori and the a posteriori estimate of A at time k, respectively.
α	The fading memory coefficient.
Q, R	The system and measurement noise covariance matrices, respectively.
η	The uncertainity vector
$\bar{A},\;\tilde{A}$	The error in A.
E(a)	The expectation operator of the element a.
$h,\;A_{p},\;C_{p},\;m\;$	The thermometer parameters; convective heat transfer coefficient, surface area, specific heat and mass, respectively.
$T,\;\tau ,\;T_{s}$	The tempretaure, time constant, and sampling time, respectively.
M, b, k	The spring-mass-damper parameters; mass, damping coefficient, and spring constant, respectively.
$I_{n\times n}$	The identity matrix with dimensions of n×n.
